# The efficacy and safety of ciclesonide for the treatment of perennial allergic rhinitis: a systematic review and meta-analysis^☆^

**DOI:** 10.1016/j.bjorl.2018.10.008

**Published:** 2018-11-22

**Authors:** Qi Yang, Fei Wang, Bin Li, Wenbin Wu, Dengpiao Xie, Li He, Nan Xiang, Yan Dong

**Affiliations:** a Chengdu University of Traditional Chinese Medicine, Chengdu, China; b Sichuan College of Traditional Chinese Medicine, Mianyang, China; c Hospital of Chengdu University of Traditional Chinese Medicine, Chengdu, China; d Chengdu First People's Hospital, Chengdu, China

**Keywords:** Meta-analysis, Ciclesonide, Rhinitis, Allergic, Perennial, Metanálise, Ciclesonida, Rinite, Alérgica, Perene

## Abstract

**Introduction:**

Allergic rhinitis is a chronic inflammatory disease which affects 1 out of 6 individuals. Perennial allergic rhinitis accounts for 40% of AR cases. Ciclesonide is one of the relatively new intranasal steroid for allergic rhinitis.

**Objective:**

The purpose of this study was to evaluate the efficacy and safety of ciclesonide in the treatment of perennial allergic rhinitis.

**Methods:**

We searched Pubmed, Scientific Citation Index, Embase, Clinical Trial Registries for randomized controlled trials and Cochrane Central Register of Controlled Trials to find out the randomized controlled Trial comparing ciclesonide with placebo for PAR.

**Results:**

Eight studies were included. In comparison with placebo groups, ciclesonide groups significantly decreased Reflective Total Nasal Symptom Score (MD = −0.56; 95% CI −0.72 to 0.39, *p* < 0.00001) with heterogeneity (*p* = 0.19, *I*^2^ = 24%), Instantaneous Total Nasal Symptom Score (MD = −0.57; 95% CI −0.75 to −0.39, *p* < 0.00001) with heterogeneity (*p* = 0.34, *I*^2^ = 11%). A significant effect for Reflective Nasal Symptom Score Subtotal (MD = −0.15; 95% CI −0.18 to −0.13, *p* < 0.00001) with heterogeneity (*p* = 0.12, *I*^2^ = 24%) was also demonstrated. Rhinoconjunctivitis quality of life questionnaire score (RQLQs) (MD = −0.27; 95% CI −0.39 to −0.15, *p* < 0.00001) with heterogeneity (*p* = 0.58, *I*^2^ = 0%) in the treatment of ciclesonide was also significantly reduced. In addition, the difference in Treatment-Emergent Adverse Events between the two groups was not significant.

**Conclusion:**

Ciclesonide can improve perennial allergic rhinitis without increasing adverse events. Ciclesonide may be another valuable choice for perennial allergic rhinitis in the future.

## Resumo

**Introdução:**

A rinite alérgica é uma doença inflamatória crônica que afeta um a cada seis indivíduos. A rinite alérgica perene é responsável por 40% dos casos de rinite alérgica. A ciclesonida é um dos corticosteroides intranasais mais novos para o tratamento dessa condição clínica.

**Objetivo:**

Avaliar a eficácia e segurança da ciclesonida no tratamento da rinite alérgica perene.

**Método:**

Uma busca foi feita nos bancos de dados Pubmed, *Scientific Citation Index*, Embase e *Clinical Trial Registries* por ensaios clínicos randomizados e *Cochrane Central Register of Controlled Trials* por estudos controlados randomizados que comparassem ciclesonida com placebo no tratamento da rinite alérgica perene.

**Resultados:**

Oito estudos foram incluídos. Em comparação com os grupos placebo, os grupos ciclesonida mostraram diminuição significante no escore do *Reflective Total Nasal Symptom Score* (DM = −0,56; IC 95%: −0,72 a −0,39, *p* < 0,00001) com heterogeneidade (*p* = 0,19, I^2^ = 24%), do *Instantaneous Total Nasal Symptom Score* (DM = −0,57; IC95%: −0,75 a −0,39, *p* < 0,00001) com heterogeneidade (*p* = 0,34, I^2^ = 11%). Um efeito significante no escore do *Reflective Nasal Symptom Score* Subtotal (DM = −0,15; IC 95%: −0,18 a −0,13, *p* < 0,00001) com heterogeneidade (*p* = 0,12, I^2^ = 24%) também foi demonstrado. O escore do *Rhinoconjunctivitis Quality of Life Questionnaire score* (RQLQs) (DM = −0,27; IC 95%: −0,39 a −0,15, *p* < 0,00001) com heterogeneidade (*p* = 0,58, I^2^ = 0%) também foi significantemente reduzido no tratamento com ciclesonida. Além disso, a diferença em relação aos eventos adversos emergentes do tratamento entre os dois grupos não foi significante.

**Conclusão:**

A ciclesonida pode melhorar a rinite alérgica perene sem aumentar os eventos adversos. Esse fármaco pode ser outra opção valiosa para a rinite alérgica perene no futuro.

## Introduction

Allergic Rhinitis (AR), a chronic inflammatory disease, is characterized by nasal itching, sneezing, runny nose and congestion.^1^ As a highly prevalent condition, AR affects 1 out of 6 individuals. The symptoms of AR interfere with all aspects of daily life that are associated with decreased sleep quality and performance at work.^2^

Despite currently available treatment options, the incidence of AR is increasing. It remains the leading cause of morbidity, absenteeism and restricted activities and is related to considerable cost pressures in the health care system.^3^, ^4^ AR can be divided into seasonal and perennial forms. Perennial allergic rhinitis (PAR) accounts for 40% of AR cases.^5^ AR is a Type 1 IgE-mediated hypersensitivity reaction.^6^

Intranasal corticosteroids (INS) represent the standard treatment for AR of all severities owing to their anti-inflammatory activity.^7^, ^8^ Systematic reviews and meta-analyses revealed topical corticosteroids are superior to antihistamines in putting nasal symptoms of AR under control.^9^, ^10^ Ciclesonide was approved by the FDA as one of the relatively new INS additions to the AR armamentarium in 2006.^11^

Ciclesonide in PAR have been evaluated in several randomized controlled trials (RCT). However, the evidence from the currently available individual randomized trials concerning ciclesonide in PAR is not convincing. Whether ciclesonide has an effect on PAR and whether it plays a role in prevention and treatment remains to be seen. To figure out these issues, we engaged in a systematic review with meta-analysis of randomized controlled trials to analyze the effect of ciclesonide in the treatment of PAR.

## Methods

### Data sources

**Figure 1:**
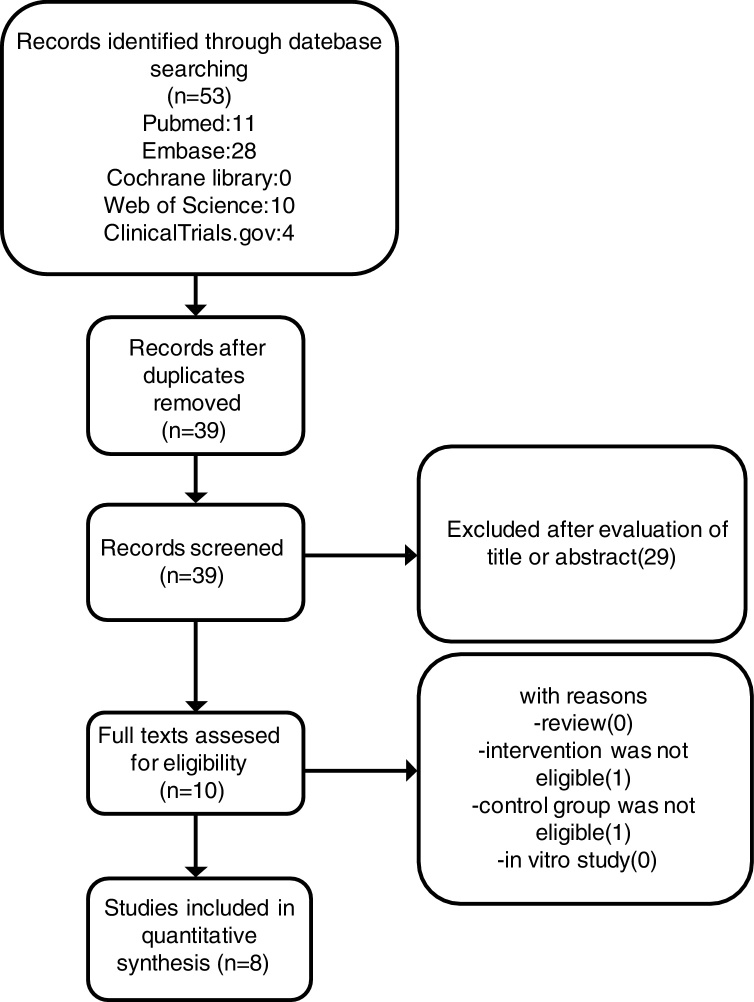
Selection of studies.

We searched Pubmed, Scientific Citation Index, Embase, Clinical Trial Registries for randomized controlled trials and Cochrane Central Register of Controlled Trials, with a search deadline of July 2018. We used the following keywords: “Rhinitis, Allergic, Perennial”, “Rhinitis, Allergic, Nonseasonal”, “Ciclesonide” and “random* controlled trial” (Fig. 1). In order to identify potentially pertinent studies, we scanned the citations of the included studies.

### Study selection

Two independent reviewers assessed the title and abstract of relevant papers. If the study was randomized trials and contrasted ciclesonide with placebo for patients with “perennial allergic rhinitis”, the study was included.

### Data extraction and quality assessment

The data information of characteristics of methods, participants, interventions and results were extracted independently by two reviewers. The Cochrane Handbook for Systematic Reviews of Interventions^12^ was used to assess the quality of included studies by evaluating the risk of bias. Any discrepancy was resolved by the third author.

### Outcome definition

The primary outcome was the change in average A.M. and P.M. reflective Total Nasal Symptom Score (rTNSS), A.M. instantaneous Total Nasal Symptom Score (iTNSS) and the second outcome included changes in average A.M. and P.M. reflective Nasal Symptom Score (rNSS), Rhinoconjunctivitis Quality of Life Questionnaire score (RQLQs). Treatment-Emergent Adverse Events (TEAEs) were used to monitor the safety.

### Data synthesis and analysis

The effect size of continuous outcomes was evaluated by weighted mean difference (WMD) and dichotomous outcomes assessed by Risk Ratio (RR) with 95% Confidence Interval (CI). Heterogeneity was evaluated with *I*^2^ statistics. A random-effects model was applied regardless of the heterogeneity of the results. Statistical assessments were performed using Review Manager, version 5.3.

## Results

### Study selection and study characteristics

In the initial search 53 related publications were identified in total. 14 duplicates were removed afterward. 29 studies were excluded by reading the title or abstract. The remaining 10 full-text articles were reviewed and 2 studies were excluded. Finally, 8 trials^13^, ^14^, ^15^, ^16^, ^17^ (NCT01451541, NCT01033825, NCT01378429) enrolling 4039 patients were included (Table 1). The selection process of studies was showed in Fig. 1. Duration of treatment was 6–52 weeks.

**Table 1:** Summary of trials included in the meta-analysis.

Study	Year	No. of patients (ciclesonide/placebo)	Interventions	Duration (weeks)	Outcomes
NCT01451541	2014	758 (511/247)	Ciclesonide 37 μg	12	rTNSS iTNSS
			Ciclesonide 74 μg		TEAE
NCT01033825	2012	160 (105/55)	Ciclesonide 160 μg	6	rTNSS iTNSS
			Ciclesonide 200 μg		rNSS
			Ciclesonide 320 μg		
Eli (13)	2007	471 (238/233)	Ciclesonide 200 μg	6	rTNSS rNSS
					RQLQ TEAE
Paul (14)	2007	663 (441/222)	Ciclesonide 200 μg	52	rTNSS rNSS
					RQLQ TEAE
William (15)	2012	1110 (803/307)	Ciclesonide 74 μg	26	rTNSS iTNSS
			Ciclesonide 148 μg		rNSS RQLQ
					TEAE
Kenneth (16)	2007	123 (81/42)	Ciclesonide 200 μg	12	rTNSS rNSS
					TEAE
NCT01378429	2014	89 (47/42)	Ciclesonide 74 μg	6	rTNSS
William (17)	2008	665 (500/165)	Ciclesonide 25 μg	12	rTNSS TEAE
			Ciclesonide 100 μg		
			Ciclesonide 200 μg		

### Quality assessment of included studies

**Figure 2:**
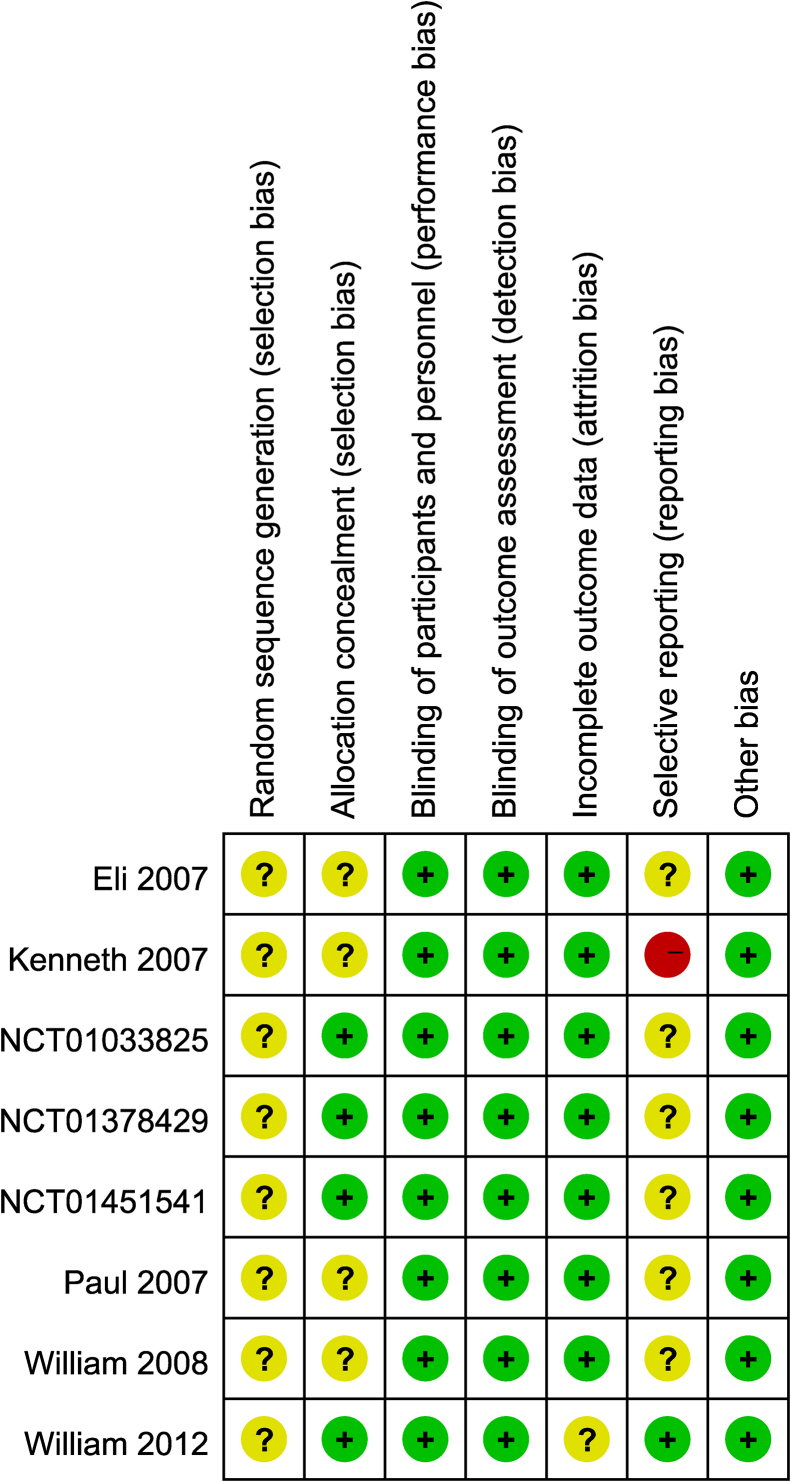
Risk of bias graph according to recommendations from the Cochrane collaboration.

All 8 studies^13^, ^14^, ^15^, ^16^, ^17^ (NCT01451541, NCT01033825, NCT01378429) were randomized and double-blind. Seven studies^13^, ^14^, ^15^, ^17^ (NCT01451541, NCT01033825, NCT01378429) were multicentre trials. However, all studies did not provide concrete randomization methods. All studies^13^, ^14^, ^15^, ^16^, ^17^ (NCT01451541, NCT01033825, NCT01378429) reported blinding of participants and personnel. All studies did not have reporting bias. Five studies reported withdrawals and one study^15^ was analyzed on an intention-to-treat basis (Fig. 2).

### Effects on rTNSS

**Figure 3:**
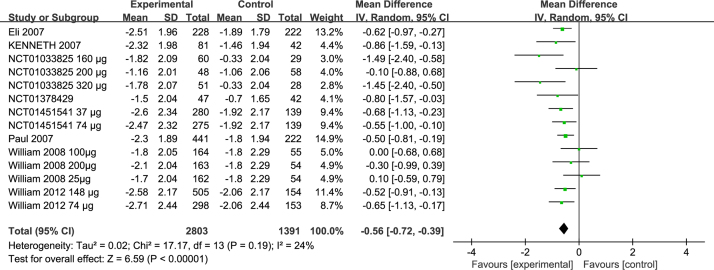
Forest plots of rTNSS of patients treated with ciclesonide.

Eight studies included the comparison of the change in rTNSS between eight groups^13^, ^14^, ^15^, ^16^, ^17^ (NCT01451541, NCT01033825, NCT01378429). The pooled result showed that there was significant difference between the two groups (MD = −0.56; 95% CI −0.72 to −0.39, *p* < 0.00001) (Fig. 3) with heterogeneity (*p* = 0.19, *I*^2^ = 24%) (Fig. 3).

### Effects on iTNSS

**Figure 4:**
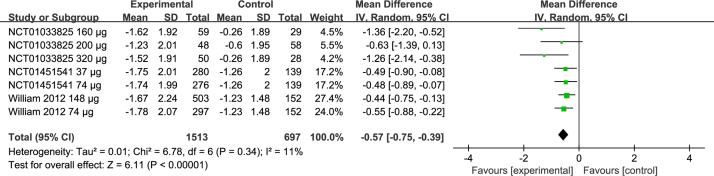
Forest plots of iTNSS of patients treated with ciclesonide.

Three studies included comparison of iTNSS between three groups^15^ (NCT01451541, NCT01033825). Pooled results showed that there was a significant difference between the two groups (MD = −0.57; 95% CI −0.75 to −0.39, *p* < 0.00001) (Fig. 4) with heterogeneity (*p* = 0.34, *I*^2^ = 11%) (Fig. 4).

### Effects on rNSS

**Figure 5:**
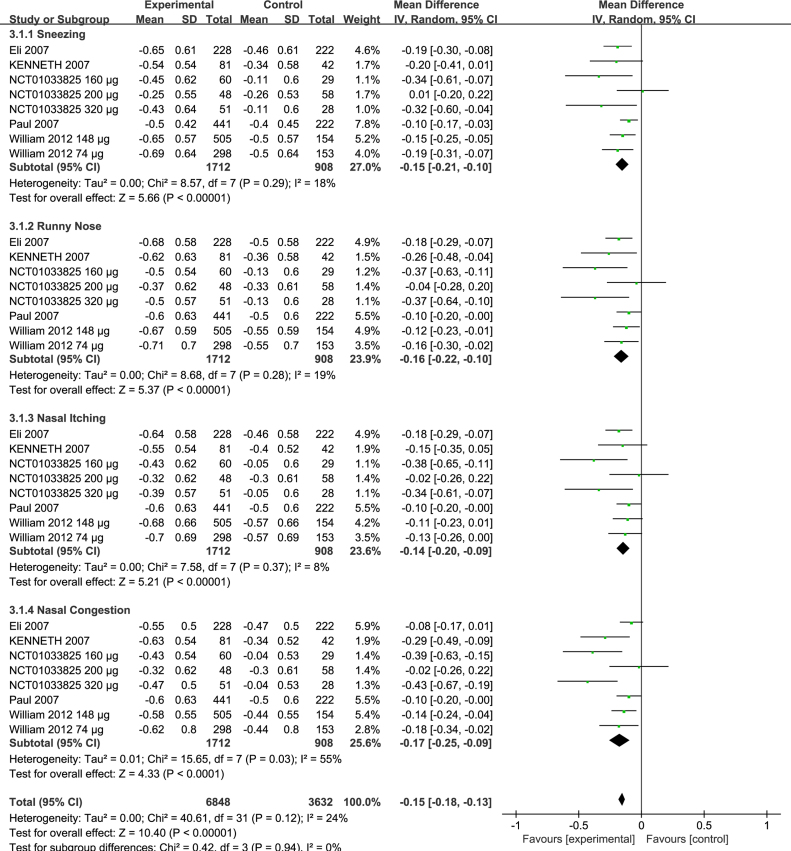
Forest plots of rNSS of patients treated with ciclesonide.

We compared rNSS in five trials^14^, ^15^, ^16^, ^17^ (NCT01033825). There was significant difference between the two groups, sneezing (MD = −0.15; 95% CI −0.21 to −0.10, *p* < 0.00001) with heterogeneity (*p* = 0.29, *I*^2^ = 18%) (Fig. 5), runny nose (MD = −0.16; 95% CI −0.22 to −0.10, *p* < 0.00001) with heterogeneity (*p* = 0.28, *I*^2^ = 19%) (Fig. 5), nasal itching (MD = −0.14; 95% CI −0.20 to −0.09, *p* < 0.00001) with heterogeneity (*p* = 0.37, *I*^2^ = 8%) (Fig. 5), nasal congestion (MD = −0.17; 95% CI −0.25 to −0.09, *p* < 0.0001) with heterogeneity (*p* = 0.03, *I*^2^ = 55%) (Fig. 5), Subtotal (MD = −0.15; 95% CI −0.18 to −0.13, *p* < 0.00001) with heterogeneity (*p* = 0.12, *I*^2^ = 24%) (Fig. 5).

### Effects on RQLQs

**Figure 6:**
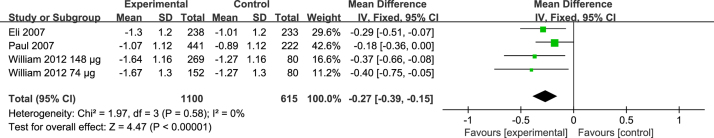
Forest plots of RQLQs of patients treated with ciclesonide.

RQLQs was compared in the three trials.^13^, ^14^, ^15^ Compared with placebo, ciclesonide significantly reduced RQLQs (MD = −0.27; 95% CI −0.39 to −0.15, *p* < 0.00001) with heterogeneity (*p* = 0.58, *I*^2^ = 0%) (Fig. 6).

### Safety

**Figure 7:**
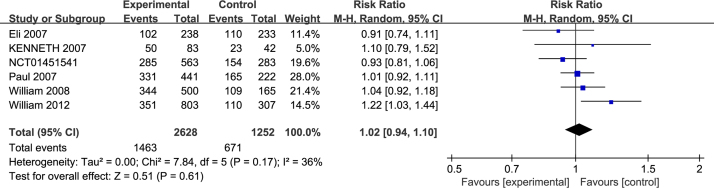
Forest plots of TEAEs of patients treated with ciclesonide.

Safety was assessed by monitoring TEAEs. For TEAEs, six trials^13^, ^14^, ^15^, ^16^, ^17^ (NCT01451541) reported complete data. There was no significant difference between the two groups (RR = 1.02; 95% CI 0.94–1.10, *p* = 0.61) with heterogeneity (*p* = 0.17, *I*^2^ = 36%) (Fig. 7).

### Publication bias and sensitivity analysis

There was no evidence of significant publication bias by Egger's test for rTNSS (*t* = −0.52, *p* = 0.609).

## Discussion and conclusions

Antihistamines and corticosteroids are current treatments for controlling AR symptoms. INS are the most effective available drug suppressing all rhinitis symptoms which include nasal blockage.^18^ However, although widely used, ciclesonide for AR still is lacking in clear evidence to make decisive recommendations for a therapeutic option. In the present study, we performed a search to evaluate the efficacy and safety of ciclesonide in patients with PAR. In this review, we found that the ciclesonide might be able to decrease rTNSS, iTNSS, rNSS, RQLQs without increasing TEAEs in the short term.

Ciclesonide is the latest inhaled glucocorticosteroid to treat symptoms of asthma and AR.^19^ The anti-inflammatory effect of ciclesonide is seen solely at the bronchial level, only a fraction of the drug reaching the gastrointestinal tract and becoming inactive.^20^, ^14^

In our review, ciclesonide produced significant relief in rTNSS and iTNSS. In the study by Eli,^13^ it was suggested that improvement in the rTNSS continued to increase throughout the 6 weeks of treatment. These continued improvements in nasal symptoms are associated with AR and may encourage patients to stick to treatment.

All individual rNSS declined in all patients treated with ciclesonide, especially nasal congestion, which is the most difficult symptom to treat. The overall change in rTNSS was driven by all four nasal symptoms, suggesting that all individual rNSS contributed to the overall difference between two groups. Owing to a variable degree of heterogeneity in these studies, we performed sensitivity analysis. We should treat the results cautiously, although the results did not change.

Ciclesonide produced a statistically significant reduction in RQLQs. However, studies on the clinical relevance of questionnaires showed that only 0.5 or more of the changes were clinically relevant.^21^

For TEAEs, there were no significant differences between the two groups. In our review, most of adverse events of ciclesonide were mild or moderate and well tolerated. Rates of discontinuation were similar to placebo. Typically seen Adverse Events (AEs) with INS are usually topical in nature and include nasal discomfort and nosebleeds.^22^ In the study by William,^15^ epistaxis and upper respiratory tract infection were the most commonly reported AEs. It may be as a consequence of negligible oral bioavailability (1%), high protein binding (99%) of ciclesonide and the active metabolite, with negligible impact on the hypothalamic–pituitary–adrenal axis.^23^, ^24^, ^25^

There were several limitations in our meta-analysis. First, several notable areas of variability existed in the data. The duration of intervention varied between 2 and 52 weeks and the baseline severity of the disease had some differences. Second, there is a possibility of study selection bias. Third, four of the eight studies^15^ (NCT01451541, NCT01033825, NCT01378429) were sponsored by pharmaceutical companies. We conduct a subgroup analysis by excluding these data and the results did not change.

In conclusion, ciclesonide can improve PAR without increasing adverse events. Ciclesonide may be another valuable choice for patients with PAR in the future.

## Ethical approval

This article does not contain any studies with human participants or animals performed by any of the authors.

## Informed consent

Informed consent was obtained from all individual participants included the study.

## Conflicts of interest

The authors declare no conflicts of interest.
